# Supplementary material for: Content-rich biological network constructed by mining PubMed abstracts
Source: BMC Bioinformatics. 2004 Oct 8;5:147. doi: 10.1186/1471-2105-5-147 (PMC528731; doi:10.1186/1471-2105-5-147)
Supplement: Additional File 2 — The original results of the above study (non-essential files are deleted to keep the file size under the limit set by BMC bioinformatics). [file 1471-2105-5-147-S2.bz2 › chilibotAdditionalFile2/dip05/446ID9468473E10452/html/CCR5_CCL8.html]

 


 **CCR5** and **CCL8** 
  
Found 26 abstracts in PubMed, retrieved 05.  
 

 What does Google say? 
 PDF only 
| .edu only 

---

**Interactive relationship** (e.g. stimulation, inhibition, etc)

**Stimulatory relationship**- The roles of  **CCR5**  in excitotoxic injury in  **CCR5**  deficient mice are compensated by increased CCR2 and CCR3 expression, which share the common MCP 2  [ **CCL8** ]  ligand with  **CCR5** .  Ref: 12807425 J Neurochem, 2003
**Neutral relationship**- **CCR5**  is a G protein coupled receptor for RANTES, MIP 1a, MIP 1b and MCP 2  [ **CCL8** ]  that functions as the front line coreceptor for human immunodeficiency virus type 1 infection.  Ref: 12837756 J Biol Chem< MedlineTA>J Biol Chem, 2003
- **CCR5**  is a G protein coupled receptor responding to four natural agonists, the chemokines RANTES regulated on activation normal T cell expressed and secreted, macrophage inflammatory protein MIP 1 alpha, MIP 1 beta, and monocyte chemotactic protein MCP 2  [ **CCL8** ] , and is the main co receptor for the macrophage tropic human immunodeficiency virus strains.  Ref: 12411445 J Biol Chem, 2003

**Non-interactive relationship** (e.g. studied together, co-existance, homology, etc.)

- A number of inflammatory CC chemokines, including MIP 1 alpha, MIP 1 beta, RANTES, MCP 2  [ **CCL8** ] , and HCC 1 9 74 act as  **CCR5**  agonists, while MCP 3 is a natural antagonist of the receptor.  Ref: 12402506 Receptors Channels, 2002
